# Law enforcement and mental health clinician partnerships in global mental health: outcomes for the Crisis Intervention Team (CIT) model adaptation in Liberia, West Africa

**DOI:** 10.1017/gmh.2019.31

**Published:** 2020-01-10

**Authors:** Mina Boazak, Sarah Yoss, Brandon A. Kohrt, Wilfred Gwaikolo, Pat Strode, Michael T. Compton, Janice Cooper

**Affiliations:** 1 PGY-2 Department of Psychiatry and Behavioral Sciences, Emory University School of Medicine, Atlanta, GA, USA; 2 Department of Medicine, Duke University School of Medicine; 3 The Carter Center Design, Monitoring, and Evaluation Unit; 4 Department of Psychiatry and Behavioral Sciences, George Washington University; 5 The Carter Center Mental Health Program, Liberia; 6 Georgia Public Safety Training Center, CIT International; 7 Department of Psychiatry, Columbia University College of Physicians and Surgeons; 8 The Carter Center Mental Health Program, Liberia & Department of Health Policy & Management, Rollins School of Public Health, Emory University

**Keywords:** Crisis intervention, developing countries, law enforcement, mental health, police

## Abstract

**Background:**

The Crisis Intervention Team (CIT) model is a law enforcement strategy that aims to build alliances between the law enforcement and mental health communities. Despite its success in the United States, CIT has not been used in low- and middle-income countries. This study assesses the immediate and 9-month outcomes of CIT training on trainee knowledge and attitudes.

**Methods:**

Twenty-two CIT trainees (14 law enforcement officers and eight mental health clinicians) were evaluated using pre-developed measures assessing knowledge and attitudes related to mental illness. Evaluations were conducted prior to, immediately after, and 9 months post training.

**Results:**

The CIT training produced improvements both immediately and 9 months post training in knowledge and attitudes, suggesting that CIT can benefit law enforcement officers even in extremely low-resource settings with limited specialized mental health service infrastructure.

**Conclusion:**

These findings support further exploration of the benefits of CIT in highly under-resourced settings.

## Introduction

The Crisis Intervention Team (CIT) program is a law enforcement model aimed at uniting the goals of the law enforcement and mental health communities (Compton *et al*., 2008; Watson and Fulambarker, 2012). Developed in 1988 in Memphis after a tragedy involving law enforcement and a person with mental illness, the CIT model has been used in the United States for three decades. The model operates on 10 core elements including, but not limited to, stakeholder partnerships, community ownership, training, and research evaluation (Dupont *et al*., 2007). The 10 elements are used as the guiding arms of CIT implementation and are pivotal to program success. Research on the CIT model has demonstrated positive results that include improvement in officers' knowledge, attitudes, and perceptions related to mental illnesses, both in the short- (Compton *et al*., 2008; Kohrt *et al*., 2015) and long-term (Compton and Chien, 2008; Christian Ritter *et al*., 2010), as well as improvements in officers' perceptions of the mental health system (Compton *et al*., 2008), and improvements in law enforcement behaviors related to persons with mental illnesses (Compton *et al*., 2014). These findings coincide with large-scale community uptake of this model in the United States, with recommendations that CIT should be the gold standard (Compton *et al*., 2010) for law enforcement approaches to working with individuals with mental illnesses. However, the use of CIT, or models like it, in low- and middle-income countries (LMICs) has had poor uptake and has been minimally evaluated. To date, our group only knows of the model's implementation in Liberia, with the only assessment being of a pilot 3-day training program modeled after CIT training being conducted by our group (Kohrt *et al*., 2012).

Since 2010, the Carter Center Mental Health Program (TCCMHP) has worked with the Liberia Ministry of Health to build a sustainable mental health system through workforce development, policy, and anti-stigma programming. The partnership was developed subsequent to the end of a tragic set of civil wars encompassing 14 years of the nation's history, resulting in the death of 200 000 individuals (10% of the nation's population at the start of the war) and the displacement of 1 000 000 individuals (50% of the nation's population at the start of the war). The war devastated much of Liberia's infrastructure, left many without homes, and resulted in significant levels of mental illness, with current estimates placing neuropsychiatric disorders as the seventh leading cause of disability-adjusted life years in the nation (Institute for Health Metrics and Evaluation, 2016). Facing the challenge of addressing significant mental health resource gaps, TCCMHP partnered with Liberian allies, including the Ministry of Health, to develop the nation's first mental health policy, train 281 mental health clinicians, and, as mentioned above, develop anti-stigma programs. Early in the establishment of TCCMHP, partner stakeholders voiced a need for anti-stigma efforts to include the Liberia National Police Association (LNPA). Police officers, who are often the first contacts for people with mental illness during times of crisis, were reported to retain superstitious beliefs relating to mental illness, negatively impacting the course of police calls involving mental health issues. Having identified the need, TCCMHP and ranking officials within the LNPA collaborated to determine the most appropriate evidence-based model. Ultimately, the group elected to utilize the CIT collaborative model to address their efforts. CIT experts from the United States, including a psychiatrist, a chief of police, and a family advocate, were then invited to develop and lead the first CIT-based workshop alongside Liberian partners. Based on interviews held with Liberian stakeholders, a 3-day workshop modeled after the CIT program was administered to a group of law enforcement officers and mental health clinicians. The workshop was a success, demonstrating immediate post-training improvements in knowledge, attitudes, social distance, functioning, and autonomy domains among law enforcement trainees (Kohrt *et al*., 2015). The success of the workshop led to plans for full CIT model implementation across Liberia. These were stalled due to the nation facing an Ebola outbreak in 2014 and 2015. In 2015, a full, 5-day, 40-h CIT course was developed and has since been administered on several occasions in Liberia. Anecdotally, the implementation of the CIT model in the LNPA has been a success. Stakeholder buy-in remains strong and officer interest in mental health advocacy has led to the development of the Crisis Intervention Team Association of Liberia (CITAL). Still, despite anecdotal success and the initial reported efficacy of our CIT-based workshop, questions remain regarding the efficacy of the model in Liberia. Here, we repeat our efficacy assessments on the full 5-day course of CIT trainees, assessing for immediate post-training outcomes, as well as assessing whether the CIT models' results are sustained among officers 9 months after training.

## Materials and methods

### CIT program

In Liberia, law enforcement officers, mental health clinicians, mental health advocates, and persons with mental illnesses receive CIT training. As of 2016, CIT training in Liberia was administered by a combination of American CIT experts (CIT researcher with a background in psychiatry and a mental health advocate who has administered and developed multiple CIT programs), Liberian mental health clinicians (nurses and physicians' assistants trained to independently diagnose and treat mental illnesses), and Liberian mental health advocates. After the success of the first training program, TCCMHP and LNPA conducted a training of trainers (ToT) program in 2017 in order to ensure sustainability and local ownership of the CIT program (Boazak *et al*., 2019). Since the ToT program, CIT has been led solely by Liberian personnel, including law enforcement officers’ mental health clinicians and service users.

The CIT training program is 40 hours in length and is administered over 5 days. Content includes didactic instruction defining mental illnesses commonly encountered by police officers [e.g. depression, psychosis, substance use disorders, posttraumatic stress disorder (PTSD)]; material on suicide and violence prevention; review of the mental health referral process and local resources available to officers; and coverage of verbal and non-verbal communication and verbal de-escalation techniques. The Liberian CIT program additionally covers topics contextually relevant to trainees. These include addressing misperceptions such as mental illnesses being due to demonic possession, mental illnesses being infectious, and mental illnesses being a punishment for previous misdeeds. Service users and their caregivers also play a large role in the Liberia CIT training by facilitating certain modules and sharing their recovery narratives. The CIT training attempts to evenly balance didactic instruction with practice-based exercises, focused largely on officers' de-escalation strategies. By the completion of training, trainee knowledge, perceptions, and practices toward persons with mental illnesses are expected to be more in line with the combined goals of the mental health and law enforcement communities. The training program is expected to foster partnerships and community ownership that consequently lead to policy changes, all three being on-going elements of CIT and all leading toward better outcomes for service users (Dupont *et al*., 2007). Given the challenges of measuring the aforementioned outcomes, generally, CIT program outcomes are measured through written assessments administered both prior to and at the conclusion of the CIT program.

### Assessments

To assess the immediate and 9-month follow-up outcomes of the CIT program, a combined multiple-choice/short-answer assessment was administered to officers both prior (2016-pre) to and immediately after (2016-post) a 2016 CIT training, and a final follow-up assessment was conducted prior to the start (2017-pre) of a 2017 ToT program, held 9 months later. We divide our outcomes assessments into three categories: immediate (differences between the 2016-pre and 2016-post assessments), intermediate (differences between the 2016-pre and the 2017-pre assessments), and sustaining outcomes (differences between the 2016-post and 2017-pre assessments). Data assessed were of five domains: knowledge (related to mental illnesses), attitudes (regarding mental illnesses), functioning (perceptions of the level of functioning of persons with mental illnesses), autonomy (assessments of level of autonomy that should be offered to persons with mental illnesses), and a Social Distance Scale (comfort/discomfort with social proximity to persons with mental illnesses). The knowledge and general attitude scales were developed by The Carter Center to address locally relevant myths, facts, and beliefs. The Social Distance Scale, functioning measure, and autonomy measure were adapted from the Stigma in Global Context-Mental Health Survey (SGC-MHS) which has been administered in diverse countries around the world (Pescosolido *et al*., 2013). All scored domains were rated using Likert scales, with the exception of general knowledge, which was assessed with true/false and multiple-choice questions.

### Data analysis

We analyzed outcomes only for those individuals who participated in both the 2016 CIT training and the 2017 ToT training (*n*  =  22). Data were collected and tabulated in an electronic spreadsheet, which was then imported into Matlab^©^. Participants' responses to the general knowledge domain questions were scored as the percentage of correct responses. Unanswered questions were scored as incorrect. Participants' responses for the attitudes, social distance, functioning, and autonomy domains were scored as an average of within-domain Likert scores. Group demographics were reported using count data and, where appropriate, mean values and standard deviations were reported. Outcome data were reported using mean values and standard deviations. Comparison testing was conducted using analysis of variance (ANOVA) testing. Given the group size (*n*  =  22), outcome data were reported for all trainees.

## Results

### Demographics

Of 31 individuals who participated in our 2016 CIT training, 22 (70.9%) returned for our 2017 ToT training program. The participants included 14 officers and eight mental health clinicians. All had taken the 2016-pre, 2016-post, and 2017-pre assessments.

### Immediate outcomes (2016-pre to 2016-post)

Non-significant improvements were noted across three domains (general knowledge, social distance, and autonomy) and no change or negative change in two (attitude and functioning) (Table 1).

**Table 1.** Primary analysis of participant domain scores and ANOVA *p* values

Group	Domain	2016 Pre-test		2016 Post-Test		2017 Pre-Test		ANOVA
		Score	Standard deviation	Score	Standard deviation	Score	Standard deviation	*p* value
Combined officers and clinicians	General knowledge	0.74	0.14	0.81	0.19	0.84	0.09	0.10
	Attitude	3.05	0.47	2.99	0.84	3.29	0.28	0.21
	Function	2.05	0.80	2.04	1.03	2.62	0.94	0.06
	Social distance^a^	2.04	0.94	1.6	0.80	1.64	0.80	0.18
	Autonomy	2.91	0.70	3.13	0.89	2.95	0.80	0.61

a Reduction in score represents reduced stigma.

### Intermediate outcomes (2016-pre to 2017-pre)

Four domains showed non-significant improvements in our intermediate outcomes assessments (general knowledge, attitude, social distance, and functioning) and there was a minimal, but positive change in one domain (autonomy) (Table 1).

### Sustained outcomes (2016-post to 2017-pre)

Finally, our sustained outcomes assessment revealed that some domains continued to show non-significant improvement (general knowledge, attitude, function) despite no additional intervention (Table 1).

## Discussion

Consistent with findings in the United States (Christian Ritter *et al*., 2010; Compton and Chien, 2008), our findings suggest that CIT training results in improvements in immediate and intermediate-term outcomes of CIT trainees' knowledge and attitudes relating to persons with mental illnesses. As an additional benefit to previous work (Compton and Chien, 2008), we demonstrate not just stable immediate and intermediate improvements, but our sustained outcomes assessment also demonstrated continued gains after CIT training despite no additional intervention. To our knowledge, previous studies have not demonstrated a time-influenced positive effect on CIT gains. However, caution needs to be taken in interpreting the data; given the small sample size of the training participants (*n*  =  22), none of the results demonstrated statistical significance. Further studies would need to be conducted to determine whether the results could be generalized to a larger population.

While the immediate and intermediate outcome findings were expected by our team, the sustained outcome findings were initially surprising. On evaluating the potential sustained outcomes, several factors were considered by our team. We posit that the measured continued gains in our group are a product of the mental health culture in Liberia. Liberia is a nation that struggles with mental health stigma, with many holding beliefs that demonic possession, witchcraft, and infection contribute to mental illnesses (Gwaikolo *et al*., 2017). The 2016 Global Burden of Disease results estimate mental (including self-harm and interpersonal violence) and neurologic illnesses to have prevalence rates of 57% (Evaluation IFHMA, 2016) in Liberia. One study reported depression and PTSD prevalence rates of 40% and 44%, respectively (Johnson *et al*., 2008), amongst select groups in the nation. The consequence of an increase in awareness and reduction in stigma immediately after the training may contribute to greater interaction with persons with mental illnesses, thus contributing to the continued gains observed in our sample. Furthermore, as illustrated in the 10 core elements, training only represents two of the 10 items of the CIT model. As such, it is foreseeable that while training had a clear initial effect on trainees' knowledge and attitudes, the continued gains noticed in our cohort were due to the development of partnerships between the LNPA, mental health clinicians, and mental health advocates; the ownership that the Liberian community has taken over the implementation of the CIT model (leading to the development of CITAL); the involvement and support of LNPA leadership; and the engagement with policymakers and their openness to instituting forward thinking policy changes. Finally, we cannot discount the role of selection bias influencing these findings. Of the original 2016 CIT trainees, 70.9% went on to participate in our 2017 TOT training and were included in this analysis. Participating in the follow-up training alone selects for those trainees that continue to be interested in CIT and as such are more likely to independently seek and foster relationships, activities, and behaviors that promote increasing competencies related to mental health service users.

This is uncharted territory. To our knowledge, this represents the first assessment of long-term outcomes of the CIT model in a highly under-resourced setting. While our data were non-significant, the positive findings are reassuring and point to need for further exploration. Furthermore, research is needed to evaluate changes in behavior and policing practice among CIT-trained officers. It may be that all, some, or none of the improvements observed in CIT trainees' knowledge and attitudes affect a change in law enforcement behaviors in this low-income nation. Our hypothesis is that the effect of CIT on Liberian law enforcement practices will parallel that of training on law enforcement officers in the United States. Future analysis in LMICs will need to directly assess CIT-trained *v.* non-CIT law enforcement officers' practices in the field, including officers' propensity to end calls without intervention or with arrest, diversion of individuals with mental illnesses to care where appropriate, and use of force.

## Conclusion

Our findings demonstrate that CIT training in Liberia not only results in immediate and intermediate improvements, but our sustained outcomes assessment demonstrates that domains continue to improve 9 months after training. These findings are suggestive of CIT training being of practical benefit in Liberia and potentially in other LMICs. Where a need is identified, our findings suggest that CIT training should be considered in LMICs to prepare law enforcement officers to appropriately respond to incidents involving persons with mental illnesses.
